# Transcriptomic Approach for Understanding the Adaptation of *Salmonella enterica* to Contaminated Produce

**DOI:** 10.4014/jmb.2007.07036

**Published:** 2020-08-21

**Authors:** Sojung Park, Eun woo Nam, Yeeun Kim, Seohyeon Lee, Seul I Kim, Hyunjin Yoon

**Affiliations:** 1 Department of Molecular Science and Technology, Ajou University, Suwon6499, Republic of Korea; 2 Department of Applied Chemistry and Biological Engineering, Ajou University, Suwon16499 Republic of Korea

**Keywords:** *Salmonella enterica*, produce, planktonic, epiphytic, transcriptomics

## Abstract

Salmonellosis is a form of gastroenteritis caused by *Salmonella* infection. The main transmission route of salmonellosis has been identified as poorly cooked meat and poultry products contaminated with *Salmonella*. However, in recent years, the number of outbreaks attributed to contaminated raw produce has increased dramatically. To understand how *Salmonella* adapts to produce, transcriptomic analysis was conducted on *Salmonella enterica* serovar Virchow exposed to fresh-cut radish greens. Considering the different *Salmonella* lifestyles in contact with fresh produce, such as motile and sessile lifestyles, total RNA was extracted from planktonic and epiphytic cells separately. Transcriptomic analysis of S. Virchow cells revealed different transcription profiles between lifestyles. During bacterial adaptation to fresh-cut radish greens, planktonic cells were likely to shift toward anaerobic metabolism, exploiting nitrate as an electron acceptor of anaerobic respiration, and utilizing cobalamin as a cofactor for coupled metabolic pathways. Meanwhile, *Salmonella* cells adhering to plant surfaces showed coordinated upregulation in genes associated with translation and ribosomal biogenesis, indicating dramatic cellular reprogramming in response to environmental changes. In accordance with the extensive translational response, epiphytic cells showed an increase in the transcription of genes that are important for bacterial motility, nucleotide transporter/metabolism, cell envelope biogenesis, and defense mechanisms. Intriguingly, *Salmonella* pathogenicity island (SPI)-1 and SPI-2 displayed up- and downregulation, respectively, regardless of lifestyles in contact with the radish greens, suggesting altered *Salmonella* virulence during adaptation to plant environments. This study provides molecular insights into *Salmonella* adaptation to plants as an alternative environmental reservoir.

## Introduction

Contaminated produce has frequently been reported as a source of foodborne disease outbreaks worldwide for the past two decades [1]. Because vegetables and fruits are regarded as primary components of healthy nutrition, and are commonly consumed without thermal treatments, the safety of agricultural produce is critical for preventing and controlling associated foodborne diseases. However, food hygiene is challenged by the multifaceted survival strategies of enteric pathogens. Although enteric pathogens have evolved to survive and adapt to the hostile conditions within the gastrointestinal tract of their host species, they are also able to colonize plant surfaces by forming biofilms and thereby persist for long periods, which can subsequently lead to human infections via contaminated farm produce [2, 3]. *Salmonella* can also penetrate into the interior of plant tissues, exploiting plant stomata and trichomes as entry portals, and persist in the intercellular space [4-6]. Enteric pathogens contaminate soil and irrigation water via infected animal feces and in turn contaminate farm produce, exploiting plants as a vector for transmission between animal hosts [3, 7]. However, the bacterial determinants that enable enteric pathogens to adapt to unfavorable plant environments remain unclear. Therefore, in an effort to unveil the underlying mechanism of bacterial adaptation to plants, we investigated the comprehensive transcriptome of *Salmonella enterica* in contact with raw radish greens as a model system.

*Salmonella* is a motile, gram-negative, rod-shaped genus belonging to the Enterobacteriaceae family, and one of the top 4 causes of diarrheal diseases that account for 70% of global foodborne diseases, including *Escherichia coli*, norovirus, *Campylobacter*, and *Salmonella* [8]. Salmonellosis commonly causes self-limiting gastroenteritis; however, life-threatening bacteremia, endovascular, and localized infections may occur depending on host health conditions and *Salmonella* serotypes [9]. The genus *Salmonella* consists of two species, *S. enterica* and *S. bongori*, and contains more than 2,500 different serotypes [9]. While all serotypes can cause diseases in humans, *S. enterica* serovars, including Typhimurium and Enteritidis, are the most notorious serotypes associated with *Salmonella* transmission from food products to humans [10]. The *S. enterica* serovar Virchow used in this study ranks among the five most frequent serotypes in Europe, and its prevalence in human salmonellosis has increased rapidly across all continents [11, 12]. Above all, it is notable that S. Virchow tends to cause bacteremia more frequently than S. Typhimurium and S. Enteritidis in the immunocompromised [13, 14] as well as display resistance against multiple antibiotics, leading to treatment failures [15, 16]. Poultry and poultry products are dominant sources for S. Virchow transmission to humans; thus, we used an S. Virchow strain isolated from chicken meat in the transcriptomic analysis.

Radish (*Raphanus raphanistrum* subsp. *sativus*) is consumed as an edible root vegetable all over the world, and its green leaves are used in fresh salads, pickles, and kimchi (the fermented Korean vegetable dish) in East Asia. Due to its nutritional benefits as well as high vitamin and mineral contents, radish greens are commonly eaten without cooking, and have occasionally been associated with foodborne disease outbreaks [17].

Considering the different bacterial lifestyles in fluid environments, two different models of *Salmonella* contamination were designed in radish greens: planktonic cells scavenging nutrients excreted from cut plant tissues, and epiphytic cells adhering to plant tissues and persisting for a long time. Our transcriptomic analysis on S. Virchow in contaminated raw produce contributes to a more comprehensive understanding of how enteric pathogens adapt to plants as an alternative reservoir in various environments.

## Materials and Methods

### Bacterial Strain

S. Virchow FORC_038 was isolated from chicken meat in Korea and provided by the Ministry of Food and Drug Safety in the Republic of Korea. Its whole genome was sequenced, and the genome annotation information was deposited into GenBank under the accession number CP015574.

### Bacterial Growth Conditions and Radish Greens Preparation

*Salmonella* cells were cultured in Luria-Bertani (LB) broth (BD Difco, USA) with vigorous shaking (220 rpm) at 37oC overnight (around 15 h) and washed with phosphate-buffered saline (PBS). The cells were resuspended in 100 ml PBS containing 14 g fresh-cut radish greens at a density of 2 × 10^7^ colony-forming units (CFU)/ml in a beaker and incubated statically at room temperature as described in our previous study [18]. For preparation of radish greens, the radish was washed once with 0.01% NaClO, and twice with ddH_2_O. Only the radish leaves were cut into 6 cm long pieces and added to PBS to facilitate bacterial contamination. Bacterial growth (optical density at 600 nm [OD_600_]) and viability were measured at the indicated times after inoculation. Live cells were enumerated using XLD agar (Oxoid, UK). Planktonic cells were sampled proximally to the cut plant without agitation, and used for the OD_600_ and viability measurements. For sampling epiphytic cells adhering to plant tissues, plant pieces were removed and rinsed with PBS. Adherent cells were subsequently detached by glass bead beating and plated on XLD agar. As a control to compare the bacterial responses to plants, *Salmonella* cells were treated with only PBS without plant tissues, and growth and viability were measured in parallel. For phytic acid (Sigma-Aldrich, USA) treatment, bacterial cells pre-cultured in LB broth were resuspended in PBS containing fresh-cut radish greens as described above, and different concentrations of phytic acid were added to the bacterial suspension.

### RNA Extraction

Planktonic and epiphytic cells were harvested separately as described above and immediately treated with RNAprotect Bacteria Reagent (Qiagen, Germany) to minimize RNA degradation. To counterbalance the bacterial responses to plant tissues, bacterial cells incubated only in PBS for the same times as those in contact with plants, were processed in the same manner. Bacterial total RNA was extracted using the RNeasy Mini Kit (Qiagen) according to the manufacturer’s instructions, and residual DNA was subsequently removed using the Turbo DNA-free Kit (Ambion, USA). Isolated RNA was analyzed using an Agilent 2100 Bioanalyzer (Agilent Technologies, USA), and those with RNA integrity numbers (RIN) > 7 were used for sequencing analysis [19]. Three biological replicates were used to produce RNA samples from each condition for sequencing.

### RNA Sequencing (RNA-Seq) and Analysis

Total RNA was processed with the Ribo-Zero Kit (Epicentre Biotechnologies, USA) to remove rRNA, and then subjected to mRNA-Seq library construction using the TruSeq RNA Sample Preparation Kit v2 (Illumina, USA). RNA-Seq was conducted using the Illumina Hiseq 2500 platform (Illumina) generating single-end reads approximately 100 bp in length (Chunlab, Korea). The RNA sequencing reads were mapped to the FORC_038 genome sequence (GenBank accession number CP015574) using the CLRNAseq program (Chunlab) and normalized using Reads Per Kilobase of transcript per Million mapped reads (RPKM), Relative Log Expression (RLE), and Trimmed Mean of M-value (TMM) [20-22]. To standardize mRNA abundance in response to plant tissues, log_2_[FC] (log_2_[fold change between the RNA samples]) values were computed by dividing the TMM-normalized reads of planktonic or epiphytic cells by the TMM-normalized reads of PBS-treated cells. Since the TMM method showed a coefficient of variation (CV) value that was lower than those by the RPKM and RLE methods, the TMM method was applied for the standardization of mRNA transcription levels. For statistical analysis, to determine differential expression of the RNA-Seq results, the empirical analysis of digital gene expression in R (edgeR; The R Foundation,) package was employed [23]. Genes with false discovery rate values < 0.05 were first chosen in edgeR analysis, and those with log_2_[FC] values > 3 or < -3 were then determined as differentially expressed genes (DEGs) in contact with the radish greens. For comparison of transcriptomic profiles between plant contact conditions, RNA-Seq results were visualized using scatter plots by the CLRNASeq program. DEGs were classified using Clusters of Orthologous Groups (COG) analysis [24] based on predicted functions of the encoded products, and their expression levels were displayed using heat maps generated by Gitools v2.2.2 [25].

### Real-Time Reverse Transcription Polymerase Chain Reaction Analysis (qRT-PCR)

Bacterial RNA was extracted and treated with DNase as described above. Purified RNA was subjected to cDNA synthesis using the RNA to cDNA EcoDry Premix (Takara, Japan). qRT-PCR was conducted using the StepOnePlus Real-Time PCR system (Applied Biosystems, USA) with Power SYBR Green PCR Master Mix (Applied Biosystems). The qRT-PCR primers were designed using Primer Express Software ver. 3.0 (Applied Biosystems) and are listed in Table S1. The mRNA expression levels of test genes were normalized using those of *gyrB* [26]. qRT-PCR was performed using RNA samples isolated from three different biological replicates, and the data were presented as the mean values ± standard deviations. The Student’s *t*-test was used to produce p-values for determining the significance.

## Results and Discussion

### Effect of Radish Greens on *Salmonella* Viability

**Fig. 1:**
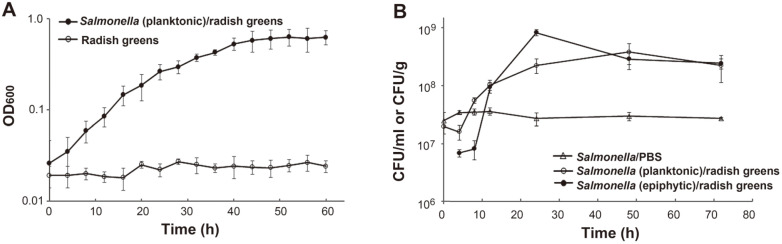
Growth of *Salmonella enterica* serovar Virchow FORC_038 in contact with radish greens. (**A**) Bacterial growth curve in the presence of radish greens. *Salmonella* cells were added to phosphate-buffered saline (PBS) containing fresh-cut radish greens at 2 × 10^7^ colony-forming units (CFU)/ml and the optical density at 600 nm (OD_600_) was measured. PBS containing only radish greens was used a control to examine the contamination with indigenous bacteria. The data from three independent tests were averaged. (**B**) Bacterial enumeration in the presence of radish greens. The numbers of planktonic cells (CFU/ml) and epiphytic cells (CFU/g) adhering to plant tissues were counted using XLD agar plating. Bacterial viability in PBS was measured as a control. The average values of three biological replicates were plotted.

It is known that enteric pathogens, such as *Salmonella*, can persist and proliferate in unfavorable plant environments by scavenging for nutrients excreted from damaged plant tissues [18, 27]. S. Virchow FORC_038 also showed growth increases in the presence of radish greens (Fig. 1A). In accordance with the incremental bacterial growth in contact with plant tissues, the viability of planktonic bacteria also increased, displaying an approximately 20-fold increased population at 48 h after inoculation (Fig. 1B). The numbers of *Salmonella* cells adhering to plant tissues rapidly increased around 12 h and peaked at 24 h post-inoculation. To differentiate bacterial transcriptional responses between lifestyles, bacterial RNA was harvested from planktonic and sessile cells at 24 h post-inoculation and subjected to transcriptomic analysis.

### Comprehensive Gene Expression Profiling in Contact with Radish Greens

**Fig. 2:**
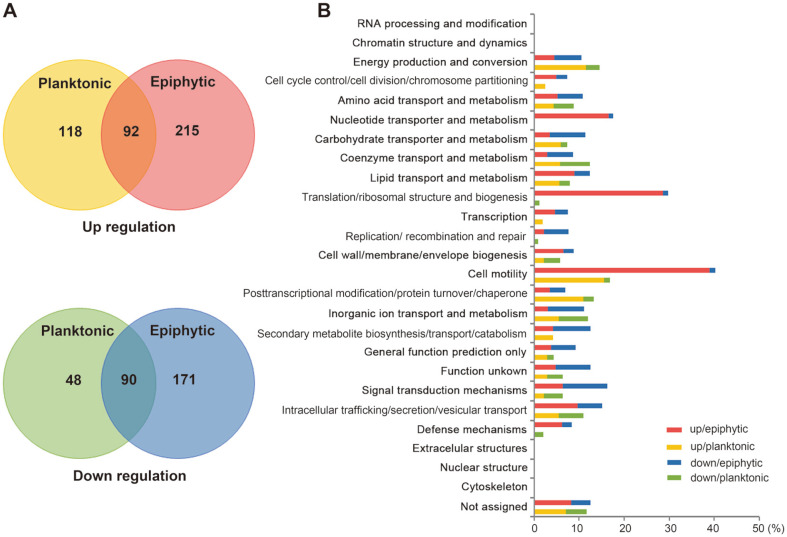
Characterization of differentially regulated genes (DEGs). (**A**) Numbers of DEGs that were up- or downregulated in planktonic or epiphytic cells in contact with radish greens. Genes with altered transcription of three-fold or more in the presence of plants were counted, and the numbers are shown in Venn diagrams. (**B**) Functional categorization of DEGs. Genes that were up- or downregulated three-fold or more in the presence of plants were grouped based on Clusters of Orthologous Groups (COG) analysis, and their percentages in each COG group are illustrated using bars of different colors depending on lifestyles.

Relative to bacterial cells not treated with radish greens, DEGs with differential expressions of log_2_[FC] > 3 or < -3 were selected in planktonic and epiphytic cells (Table S2). Planktonic cells contained 210 upregulated genes and 138 downregulated genes while epiphytic cells had 307 upregulated genes and 261 downregulated genes (Fig. 2A). A total of 182 DEGs were observed in common between planktonic and epiphytic cells, including 92 upregulated and 90 downregulated genes. DEGs were categorized based on their predicted functions using the COG analysis (Fig. 2B). *Salmonella* adhering to plant tissues exhibited remarkable transcriptional alterations in genes associated with cell motility (40.26%), translation/ribosomal structure and biogenesis (29.82%), and nucleotide transport and metabolism (17.58%), and most genes in these three groups were significantly upregulated. This result suggests that *Salmonella* experienced dynamic environmental changes on the surfaces of plant tissues and produced more proteins to rapidly adapt to a new niche and interact with the environment. Aside from these three groups, diverse groups including cell cycle control/cell division/chromosome partitioning (7.32% vs. 2.44%), transcription (7.43% vs. 1.86%), replication/recombination and repair (7.59% vs. 0.89%), secondary metabolite biosynthesis/transport/catabolism (12.50% vs. 4.17%), signal transduction mechanisms (16.31% vs. 6.38%), and defense mechanisms (8.33% vs. 2.08%) showed greater transcriptional alterations in epiphytic cells compared to planktonic cells. Planktonic cells showed moderate transcriptional changes in each functional group; however, the genes of three groups, including energy production/conversion, carbohydrate transporter/metabolism, and posttranscriptional modification/protein turnover/chaperone, displayed significant transcriptional increases in planktonic cells.

### Genes with Significant Transcriptional Changes in Response to Radish Greens

DEGs observed in both planktonic and epiphytic cells may play important roles for *Salmonella* to adapt to plant environments. These co-regulated genes are listed in Table S2. The 92 upregulated genes in both conditions included genes encoding flagella, siderophores, and proteins responsible for bacterial resistance. Considering the presumable abundance of plant-derived nutrients like iron, *Salmonella* cells may exploit flagella machinery and siderophore compartments to rummage for available nutrients. Genes (FORC38_4145, FORC38_4149, FORC38_4150, and FORC38_4151) predicted to produce the arginine deiminase (ADI) system were significantly upregulated in contact with radish greens, regardless of lifestyles. The ADI system provides cellular energy under oxygen-limited conditions by converting L-arginine to ornithine, ammonia, and carbon dioxide and its role has also been implicated in alleviating acidic stressors [28, 29]. Choi *et al*. has demonstrated that *Salmonella* required ADI system for its survival inside phagocytic cells of acidic pH [30]. _L_-Ascorbate, which promotes bacterial resistance against reactive oxygen species (ROS), is acquired by the ascorbate-specific phosphotransferase system encoded by the *ulaABC* operon under anaerobic conditions and processed by other *ula* regulon-encoded components [31]. Five genes of the *ula* regulon were simultaneously induced in planktonic and epiphytic cells. Furthermore, *terBCD* genes were also significantly upregulated in both cell types. These genes confer bacterial resistance to tellurite, which is responsible for cytoplasmic ROS production [32].

**Fig. 3:**
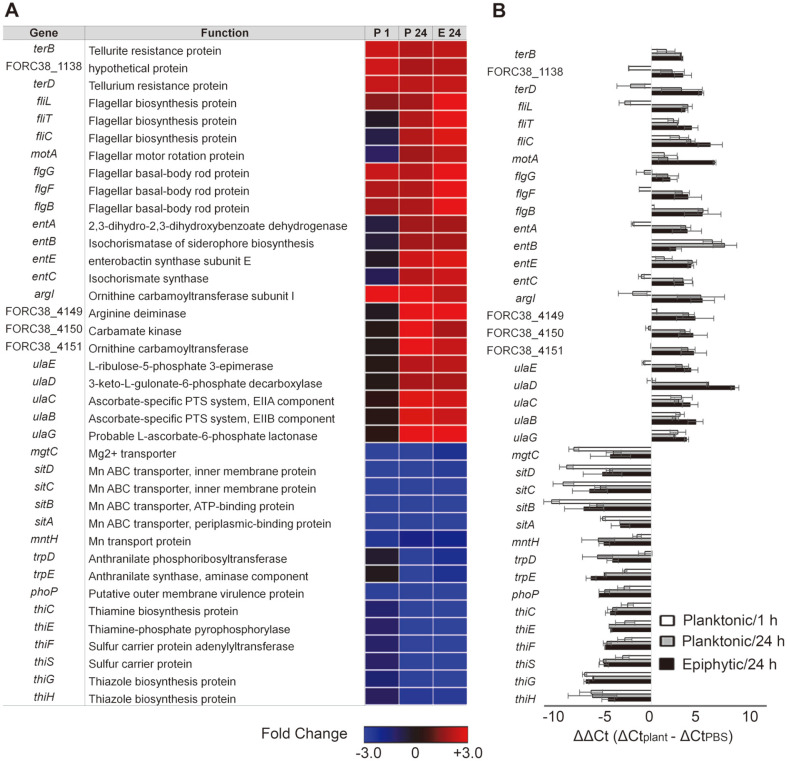
DEGs observed in both planktonic and epiphytic cells at 24 h post-contact with plants. (**A**) Heat maps of genes that were up- or downregulated in both lifestyles were represented using fold-changes (log_2_[TMM(plant)/TMM(PBS)]) and depicted using a colorimetric gradient: downregulation in blue and upregulation in red. P 1, planktonic 1 h; P 24, planktonic 24 h; E 24, epiphytic 24 h; TMM, Trimmed Mean of M-value. (**B**) Validation of DEGs using quantitative reverse transcription polymerase chain reaction (qRT-PCR). For each gene, the ΔCt values of PBS-treated cells were subtracted from the ΔCt values of planktonic or epiphytic cells, and the mean values of three independent tests were plotted with their standard deviations (SDs).

The 90 genes that were downregulated in both cell types included several genes associated with the manganese transport system and thiamine pyrophosphate (TPP) synthesis. Manganese and iron are necessary for bacterial energy metabolism and ROS resistance [33]. *Salmonella* possesses multifaceted transport systems, such as SitABCD and MntH, both of which prefer manganese to iron [34, 35]. Transcription levels of the *sitABCD* and *mntH* genes were remarkably decreased following contact with radish greens, which may be attributable to the affordable manganese discharged from plant tissues. Thiamine (Vitamin B_1_) is also a plentiful soluble nutrient present in plant tissues, and its diphosphate form, TPP, serves as a cofactor for a number of essential cellular enzymes [36]. *Salmonella* may downregulate multiple genes required for TPP biosynthesis due to an abundance of thiamine from plant tissues [37]. In addition, *mgtC* and its transcriptional regulator *phoP* were downregulated in the presence of radish greens. MgtC couples proton translocation to ATP synthesis and its absence increases cellular ATP levels [38]. Coordinated downregulation of *mgtC* and *phoP* may influence *Salmonella* energy production after contact with plants. The transcriptional expression of these co-regulated DEGs in both cell types was validated using quantitative RT-PCR (Fig. 3).

### Altered Energy Production in Planktonic Cells

**Fig. 4:**
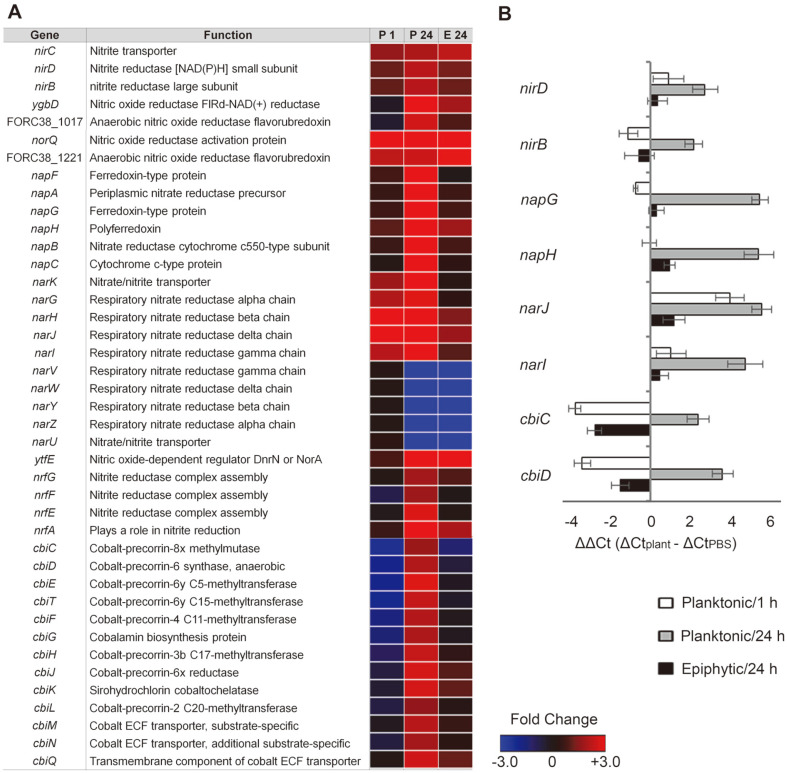
Expression of genes associated with anaerobic respiration in contact with plants. (**A**) Heat maps of genes associated with nitrate/nitrite reductase activity and cobalamin biosynthesis were represented using fold-changes (log_2_[TMM(plant)/TMM(PBS)]) and depicted using a colorimetric gradient: downregulation in blue and upregulation in red. P 1, planktonic 1 h; P 24, planktonic 24 h; E 24, epiphytic 24 h; TMM, Trimmed Mean of M-value. (**B**) Validation of DEGs using qRT-PCR. For each gene, the ΔCt values of PBS-treated cells were subtracted from the ΔCt values of planktonic or epiphytic cells, and the mean values of three independent tests were plotted with their SDs.

Transcriptome analysis revealed that the transcription of genes associated with anaerobic respiration system was significantly increased in planktonic cells. Facultative anaerobes, such as *Salmonella* spp. and *E. coli*, exploit nitrate as a terminal electron acceptor in their electron transport chain under low oxygen conditions. Genome analysis shows that S. Virchow FORC_038 contains three genetic loci relevant to nitrate reductases, including *narGHJI*, *narZYWV*, and *napABC* genes. Nitrate is abundant in many environments, and bacteria residing in the plant environment can utilize plant-derived nitrate as a source of ammonium for bacterial biosynthesis [39] or an alternative electron acceptor for energy generation [40]. The *narGHJI* and *narZYWV* operons produce distinct membrane-bound proton-translocating nitrate reductases, but nitrate reductase A, encoded by *narGHJI* is the main enzyme responsible for energy production in anaerobic environments, whereas nitrate reductase Z, encoded by *narZYWV* is known to be synthesized constitutively at low levels. The *napABC* operon encodes a periplasmic nitrate reductase, but is unlikely to generate a net proton gradient during respiration due to its localization [41]. Although the physiological role of the periplasmic nitrate reductase is yet to be defined, the expression of *napABC* genes is induced in response to anaerobic growth conditions, and *Salmonella* lacking the periplasmic nitrate reductase displayed reduced survival in the murine cecum with low oxygen and nitrate concentrations [42]. S. Virchow FORC_038 in contact with radish greens decreased the expression of the *narZYWV* operon regardless of lifestyle between planktonic and sessile cells, but remarkably increased the transcription of *narGHJI* and *napABC* operons in its planktonic status, implicating anaerobic nitrate respiration for energy production in planktonic cells (Fig. 4A). Genes required for nitrite reduction were accordingly induced in planktonic cells to detoxify or further reduce nitrite to ammonia, including the genes of *nrfABCDEGG* and nirBDC operons. As a control to understand *Salmonella* adaptation to plants, planktonic cells in contact with radish greens for 1 h were subjected to transcriptome analysis in parallel. This altered energy production pathway to anaerobic respiration suggests that *Salmonella* can harness different metabolites, such as nitrates and nitrites, to increase its fitness in low oxygen conditions due to dense bacterial populations. The altered expression of genes associated with anaerobic respiration was validated using qRT-PCR (Fig. 4B). In accordance with the anaerobic respiration of planktonic *Salmonella* in contact with plants, the genes required for cobalamin (vitamin B_12_) production showed increases in their transcription. *Salmonella* spp. synthesize cobalamin *de novo* during anaerobiosis and utilize it as a cofactor in various metabolic pathways, including methionine synthesis, ethanolamine cleavage to a carbon and/or nitrogen source, propanediol degradation to a carbon and energy source, and queuosine synthesis [43].

### Altered Translation and Ribosomal Biogenesis in Adherent Cells

**Fig. 5:**
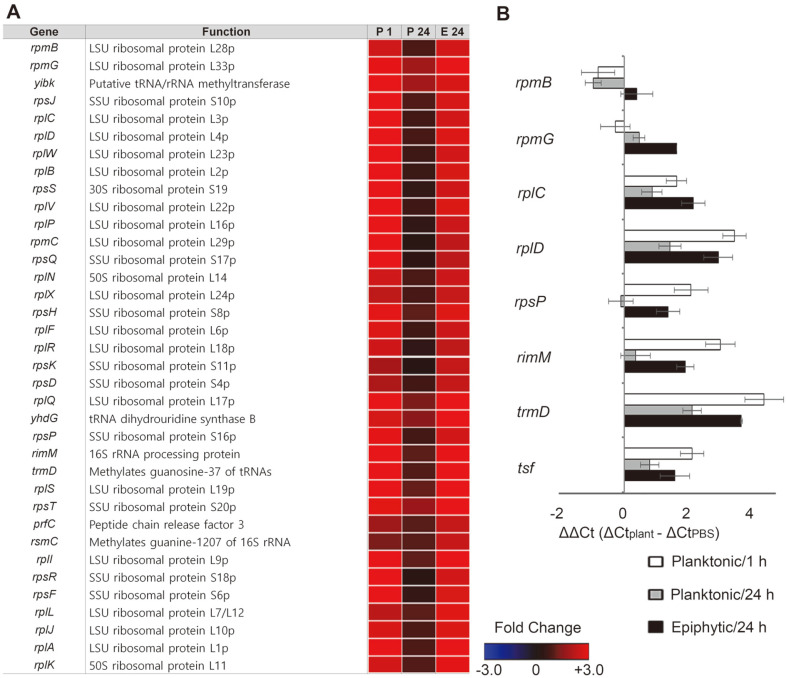
Expression of genes associated with ribosomal biogenesis in contact with plants. (**A**) Heat maps of genes associated with the bacterial translation process were represented using fold-changes (log_2_[TMM(plant)/TMM(PBS)]) and depicted using a colorimetric gradient: downregulation in blue and upregulation in red. P 1, planktonic 1 h; P 24, planktonic 24 h; E 24, epiphytic 24 h; TMM, Trimmed Mean of M-value. (**B**) Validation of DEGs using qRT-PCR. For each gene, the ΔCt values of PBS-treated cells were subtracted from the ΔCt values of planktonic or epiphytic cells, and the mean values of three independent tests were plotted with their SDs.

Bacteria that sense environmental changes reshape their cellular transcriptome and proteome to adapt to new environments. *Salmonella* under nutrient depletion conditions, for instance, employs (p)ppGpp as a second messenger to coordinate the selectivity of RNA polymerase and the activity of proteins or regulatory RNAs that are critical for stress resistance [44]. Biofilm formation stimulated by starvation and physical/chemical stressors is partly attributable to (p)ppGpp-mediated stringent responses [45]. (p)ppGpp accumulated in amino acid deprivation conditions inhibits the transcription of genes encoding ribosomal proteins and triggers the degradation of ribosomal proteins to free amino acids [44]. We observed that *Salmonella* adhering to plant tissues increased the transcription of genes associated with biofilm formation, including genes required for the production of fimbria, flagella, colonic acid, and lipopolysaccharides (Table S2). However, intriguingly, genes involved in bacterial translation processes, including ribosomal protein synthesis, elongation factor synthesis, rRNA methylation/processing, tRNA modification, and ribosomal termination complex formation, showed coordinated increases in their expression levels in epiphytic bacteria (Fig. 5A), and their transcription levels were validated using qRT-PCR (Fig. 5B). Considering the increased bacterial growth in the presence of radish greens, *Salmonella* adhering to plant tissues was less likely to suffer from nutrient starvation, but instead may have executed dramatic cellular reprogramming to establish successful colonization. S. Typhi, which causes human-specific systemic infection, also upregulated the expression of multiple ribosomal subunit genes during biofilm formation in a gallbladder-mimicking environment [46].

### Differential Expression of Virulence Genes in Contact with Radish Greens

**Fig. 6:**
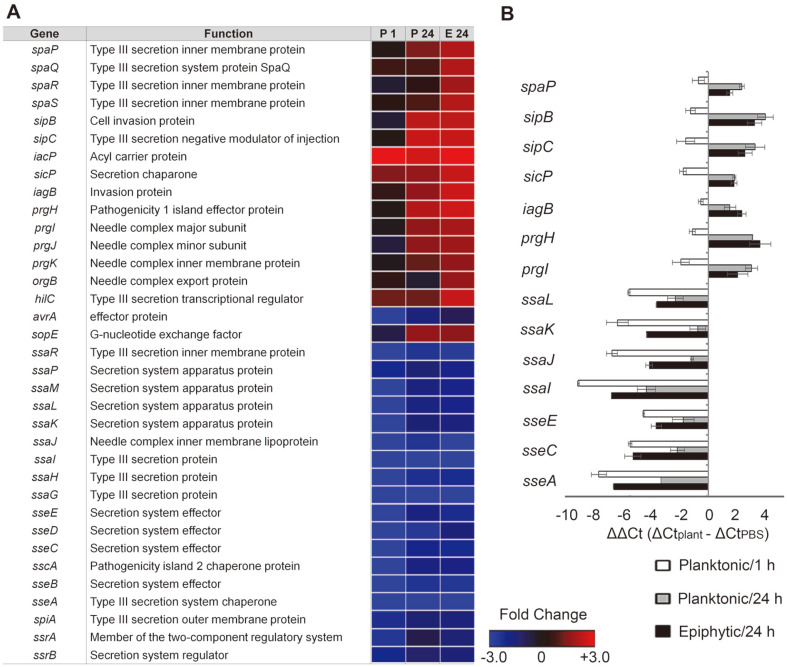
Expression of genes associated with *Salmonella* pathogenicity island (SPI)-1 and SPI-2 type III secretion systems (T3SSs) in contact with plants. (**A**) Heat maps of genes associated with SPI-1/SPI-2 T3SSs were represented using fold-changes (log_2_[TMM(plant)/TMM(PBS)]) and depicted using a colorimetric gradient: downregulation in blue and upregulation in red. P 1, planktonic 1 h; P 24, planktonic 24 h; E 24, epiphytic 24 h; TMM, Trimmed Mean of Mvalue. (**B**) Validation of DEGs using qRT-PCR. For each gene, the ΔCt values of PBS-treated cells were subtracted from the ΔCt values of planktonic or epiphytic cells, and the mean values of three independent tests were plotted with their SDs.

*Salmonella* spp. exploit two different type III secretion systems (T3SSs) as prominent virulence determinants during host animal infection. Virulence effectors translocated to host cells via *Salmonella* pathogenicity island (SPI)-1-encoded T3SS manipulate host cellular signaling and mainly stimulate *Salmonella* invasion into host cells while those translocated by SPI-2 T3SS enable *Salmonella* survival and replication inside host cells [47, 48]. Recent studies have shown that *Salmonella* mutants defective in T3SSs failed to colonize plant tissues and compromise the plant immune systems [5, 49], suggesting an important role of T3SSs in *Salmonella* adaptation to plant environments. In our previous study, we observed that S. Enteritidis and S. Typhimurium strains exposed to (napa) cabbage for 3 h displayed increased transcription of SPI-1 genes [50]. As stated previously, the S. Virchow FORC_038 strain also presented higher expression levels of genes encoding SPI-1 T3SS in contact with radish greens (Fig. 6A). Relative to planktonic *Salmonella* at 1 or 24 h, epiphytic cells adhering to plant tissues showed greater increases in the transcription of SPI-1 genes. However, SPI-2 T3SS genes were downregulated in both planktonic and epiphytic cells. Their expression was reexamined using qRT-PCR (Fig. 6B). Considering the ability of *Salmonella* to invade plant tissues and cells [4, 51], the bacteria may harness SPI-1 T3SS to access plant tissues and cells as occurs in animal infection models.

### Effects of Phytic Acid on Controlling *Salmonella* in Plants

**Fig. 7:**
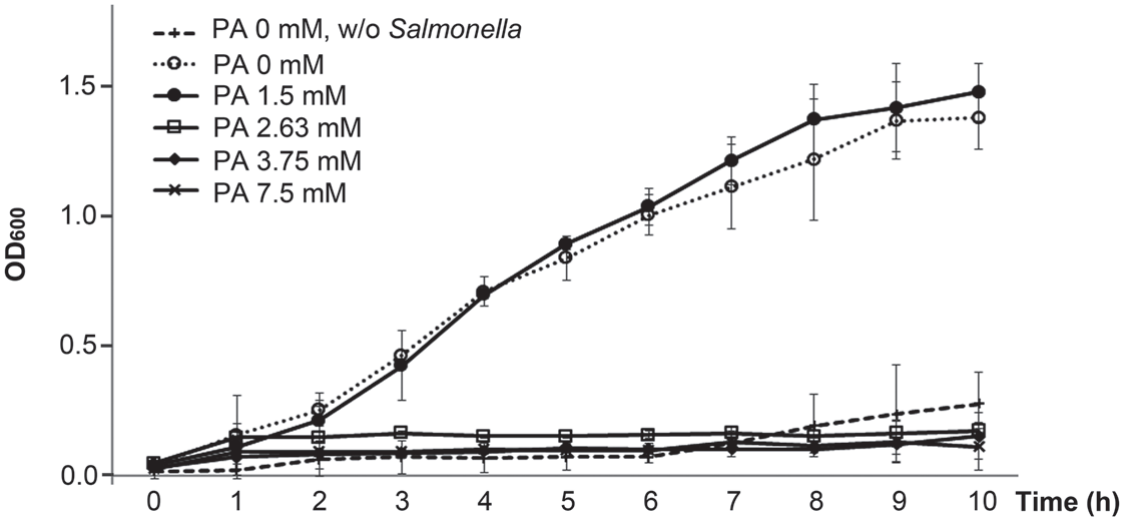
Effect of phytic acid on *Salmonella* growth in contact with plants. PBS solutions containing radish greens were supplemented with different concentrations of phytic acid from 0 mM to 7.5 mM and inoculated with S. Virchow FORC_038. Bacterial growth was measured every hour, and the OD_600_ values were averaged from three independent tests. PBS containing only radish greens without *Salmonella* was tested in parallel as a control to examine the contamination with indigenous bacteria.

Cumulative data suggest that *Salmonella* spp. exploit plants as an alternative niche for their persistence in hostile environments and as a vehicle for their transmission to preferable animal hosts [3, 7]. We observed that the presence of plant tissues stimulated *Salmonella* growth, and the transcriptome analysis suggested that *Salmonella* could utilize diverse transport systems to acquire nutrients excreted from plant tissues and employ alternative energy production pathways to cope with altered environmental conditions. As a countermeasure to control *Salmonella* proliferation in contact with plant tissues, the antimicrobial activity of phytic acid (2,3,4,5,6-pentaphosphonooxycyclohexyl dihydrogen phosphate) was tested. Phytic acid forms diverse phytates by chelating positively charged mineral ions [52], which may impede the ability of *Salmonella* to acquire plant-derived nutrients. We observed that 2.63 mM phytic acid completely abolished *Salmonella* growth even in the presence of radish greens (Fig. 7). Phytic acid has been regarded as an antinutritional component in grains and legumes, and has been used as a food additive among the Generally Recognized As Safe (GRAS) substances approved by United States Food and Drug Administration. However, the mechanism underlying antimicrobial activity of phytic acid remains unclear. Phytic acid my prevent bacteria from scavenging environmental nutrients by strongly chelating amino acids and minerals of multivalent cations, such as Fe^3+^, Ca^2+^, and Mn^2+^ [53]. The interaction between phytic acid and divalent cations adjacent to the bacterial outer membrane may further destabilize the bacterial envelope structure integrity [53]. Phytic acid, which has a wide pK_a_ range of 1.9 - 9.5, may also participate in killing activities like other acidic reagents [53].

In this study, transcriptome analysis provided a comprehensive understanding of *Salmonella* adaptation to plant environments. *Salmonella* reprogrammed the transcription of a myriad of genes in contact with plant tissues, and the repertoire of genes with altered expression levels was influenced by the bacterial lifestyles between planktonic and epiphytic types. Understanding the comprehensive transcriptional response of *Salmonella* to plants also suggests a plausible method to dampen bacterial proliferation in the presence of plant tissues.

## Supplemental Materials





Supplementary data for this paper are available on-line only at http://jmb.or.kr.

## References

[1] Bennett SD, Sodha SV, Ayers TL, Lynch MF, Gould LH, Tauxe RV (2018). Produce-associated foodborne disease outbreaks, USA, 1998-2013. Epidemiol. Infect..

[2] Bogino PC, Oliva Mde L, Sorroche FG, Giordano W (2013). The role of bacterial biofilms and surface components in plant-bacterial associations. Int. J. Mol. Sci..

[3] Wiedemann A, Virlogeux-Payant I, Chausse AM, Schikora A, Velge P (2014). Interactions of *Salmonella* with animals and plants. Front. Microbiol..

[4] Kroupitski Y, Golberg D, Belausov E, Pinto R, Swartzberg D, Granot D (2009). Internalization of *Salmonella enterica* in leaves is induced by light and involves chemotaxis and penetration through open stomata. Appl. Environ. Microbiol..

[5] Schikora A, Virlogeux-Payant I, Bueso E, Garcia AV, Nilau T, Charrier A (2011). Conservation of *Salmonella* infection mechanisms in plants and animals. PLoS One.

[6] Barak JD, Kramer LC, Hao LY (2011). Colonization of tomato plants by *Salmonella enterica* is cultivar dependent, and type 1 trichomes are preferred colonization sites. Appl. Environ. Microbiol..

[7] Kljujev I, Raicevic V, Vujovic B, Rothballer M, Schmid M (2018). Salmonella as an endophytic colonizer of plants - A risk for health safety vegetable production. Microb. Pathog..

[8] Havelaar AH, Kirk MD, Torgerson PR, Gibb HJ, Hald T, Lake RJ (2015). World health organization global estimates and regional comparisons of the burden of foodborne disease in 2010. PLoS Med..

[9] Crum-Cianflone NF (2008). Salmonellosis and the gastrointestinal tract: more than just peanut butter. Curr. Gastroenterol. Rep..

[10] Jackson BR, Griffin PM, Cole D, Walsh KA, Chai SJ (2013). Outbreak-associated *Salmonella enterica* serotypes and food Commodities, United States, 1998-2008. Emerg. Infect. Dis..

[11] Hendriksen RS, Vieira AR, Karlsmose S, Lo Fo Wong DM, Jensen AB, Wegener HC (2011). Global monitoring of *Salmonella* serovar distribution from the World Health Organization Global Foodborne Infections Network Country Data Bank: results of quality assured laboratories from 2001 to 2007. Foodborne Pathog. Dis..

[12] Olsen SJ, Bishop R, Brenner FW, Roels TH, Bean N, Tauxe RV (2001). The changing epidemiology of salmonella: trends in serotypes isolated from humans in the United States, 1987-1997. J. Infect. Dis..

[13] Ispahani P, Slack RC (2000). Enteric fever and other extraintestinal salmonellosis in University Hospital, Nottingham, UK, between 1980 and 1997. Eur. J. Clin. Microbiol. Infect. Dis..

[14] Matheson N, Kingsley RA, Sturgess K, Aliyu SH, Wain J, Dougan G (2010). Ten years experience of *Salmonella* infections in Cambridge, UK. J. Infect..

[15] Weinberger M, Solnik-Isaac H, Shachar D, Reisfeld A, Valinsky L, Andorn N (2006). Salmonella enterica serotype Virchow: epidemiology, resistance patterns and molecular characterisation of an invasive *Salmonella* serotype in Israel. Clin. Microbiol. Infect..

[16] Bertrand S, Weill FX, Cloeckaert A, Vrints M, Mairiaux E, Praud K (2006). Clonal emergence of extended-spectrum beta-lactamase (CTX-M-2)-producing *Salmonella enterica* serovar Virchow isolates with reduced susceptibilities to ciprofloxacin among poultry and humans in Belgium and France (2000 to 2003). J. Clin. Microbiol..

[17] Cho SH, Kim J, Oh KH, Hu JK, Seo J, Oh SS (2014). Outbreak of enterotoxigenic *Escherichia coli* O169 enteritis in schoolchildren associated with consumption of kimchi, Republic of Korea, 2012. Epidemiol. Infect..

[18] Kim SI, Yoon H (2019). Roles of YcfR in Biofilm Formation in *Salmonella* Typhimurium ATCC 14028. Mol. Plant Microbe. Interact..

[19] Schroeder A, Mueller O, Stocker S, Salowsky R, Leiber M, Gassmann M (2006). The RIN: an RNA integrity number for assigning integrity values to RNA measurements. BMC Mol. Biol..

[20] Risso D, Ngai J, Speed TP, Dudoit S (2014). Normalization of RNA-seq data using factor analysis of control genes or samples. Nat. Biotechnol..

[21] Robinson MD, Oshlack A (2010). A scaling normalization method for differential expression analysis of RNA-seq data. Genome Biol..

[22] Dillies MA, Rau A, Aubert J, Hennequet-Antier C, Jeanmougin M, Servant N (2013). A comprehensive evaluation of normalization methods for Illumina high-throughput RNA sequencing data analysis. Brief. Bioinform..

[23] Robinson MD, McCarthy DJ, Smyth GK (2010). edgeR: a Bioconductor package for differential expression analysis of digital gene expression data. Bioinformatics.

[24] Tatusov RL, Koonin EV, Lipman DJ (1997). A genomic perspective on protein families. Science.

[25] Perez-Llamas C, Lopez-Bigas N (2011). Gitools: analysis and visualisation of genomic data using interactive heat-maps. PLoS One.

[26] Ban GH, Kang DH, Yoon H (2015). Transcriptional response of selected genes of *Salmonella enterica* serovar Typhimurium biofilm cells during inactivation by superheated steam. Int. J. Food Microbiol..

[27] Cooley M, Carychao D, Crawford-Miksza L, Jay MT, Myers C, Rose C (2007). Incidence and tracking of *Escherichia coli* O157:H7 in a major produce production region in California. PLoS One.

[28] Casiano-Colon A, Marquis RE (1988). Role of the arginine deiminase system in protecting oral bacteria and an enzymatic basis for acid tolerance. Appl. Environ. Microbiol..

[29] Marquis RE, Bender GR, Murray DR, Wong A (1987). Arginine deiminase system and bacterial adaptation to acid environments. Appl. Environ. Microbiol..

[30] Choi Y, Choi J, Groisman EA, Kang DH, Shin D, Ryu S (2012). Expression of STM4467-encoded arginine deiminase controlled by the STM4463 regulator contributes to *Salmonella enterica* serovar Typhimurium virulence. Infect. Immun..

[31] Campos E, Montella C, Garces F, Baldoma L, Aguilar J, Badia J (2007). Aerobic L-ascorbate metabolism and associated oxidative stress in *Escherichia coli*. Microbiology.

[32] Perez JM, Calderon IL, Arenas FA, Fuentes DE, Pradenas GA, Fuentes EL (2007). Bacterial toxicity of potassium tellurite: unveiling an ancient enigma. PLoS One.

[33] Boyer E, Bergevin I, Malo D, Gros P, Cellier MF (2002). Acquisition of Mn(II) in addition to Fe(II) is required for full virulence of *Salmonella enterica* serovar Typhimurium. Infect. Immun..

[34] Kehres DG, Janakiraman A, Slauch JM, Maguire ME (2002). SitABCD is the alkaline Mn(2+) transporter of *Salmonella enterica* serovar Typhimurium. J. Bacteriol..

[35] Kehres DG, Zaharik ML, Finlay BB, Maguire ME (2000). The NRAMP proteins of *Salmonella* Typhimurium and *Escherichia coli* are selective manganese transporters involved in the response to reactive oxygen. Mol. Microbiol..

[36] Agyei-Owusu K, Leeper FJ (2009). Thiamin diphosphate in biological chemistry: analogues of thiamin diphosphate in studies of enzymes and riboswitches. FEBS J..

[37] Webb E, Febres F, Downs DM (1996). Thiamine pyrophosphate (TPP) negatively regulates transcription of some thi genes of *Salmonella* typhimurium. J. Bacteriol..

[38] Domka J, Lee J, Wood TK (2006). YliH (BssR) and YceP (BssS) regulate *Escherichia coli* K-12 biofilm formation by influencing cell signaling. Appl. Environ. Microbiol..

[39] Lin JT, Stewart V (1998). Nitrate assimilation by bacteria. Adv. Microb. Physiol..

[40] Berks BC, Ferguson SJ, Moir JW, Richardson DJ (1995). Enzymes and associated electron transport systems that catalyse the respiratory reduction of nitrogen oxides and oxyanions. Biochim. Biophys. Acta.

[41] Stewart V, Lu Y, Darwin AJ (2002). Periplasmic nitrate reductase (NapABC enzyme) supports anaerobic respiration by *Escherichia coli* K-12. J. Bacteriol..

[42] Lopez CA, Rivera-Chavez F, Byndloss MX, Baumler AJ (2015). The periplasmic nitrate reductase NapABC supports luminal growth of *Salmonella enterica* serovar Typhimurium during colitis. Infect. Immun..

[43] Roth JR, Lawrence JG, Rubenfield M, Kieffer-Higgins S, Church GM (1993). Characterization of the cobalamin (Vitamin B12) biosynthetic genes of *Salmonella* Typhimurium. J. Bacteriol..

[44] Dalebroux ZD, Swanson MS (2012). ppGpp: magic beyond RNA polymerase. Nat. Rev. Microbiol..

[45] Balzer GJ, McLean RJ (2002). The stringent response genes *relA* and *spoT* are important for *Escherichia coil* biofilms under slow-growth conditions. Can. J. Microbiol..

[46] Chin KCJ, Taylor TD, Hebrard M, Anbalagan K, Dashti MG, Phua KK (2017). Transcriptomic study of *Salmonella enterica* subspecies *enterica* serovar Typhi biofilm. BMC Genomics.

[47] Galan JE (2001). *Salmonella* interactions with host cells: type III secretion at work. Annu. Rev. Cell Dev. Biol..

[48] McGhie EJ, Brawn LC, Hume PJ, Humphreys D, Koronakis V (2009). *Salmonella* takes control: effector-driven manipulation of the host. Curr. Opin. Microbiol..

[49] Shirron N, Yaron S (2011). Active suppression of early immune response in tobacco by the human pathogen *Salmonella* Typhimurium. PLoS One.

[50] Lee H, Kim SI, Park S, Nam E, Yoon H (2018). Understanding Comprehensive tanscriptional response of *Salmonella enterica* spp. in contact with cabbage and napa cabbage. J. Microbiol. Biotechnol..

[51] Schikora A, Carreri A, Charpentier E, Hirt H (2008). The dark side of the salad: *Salmonella* Typhimurium overcomes the innate immune response of *Arabidopsis thaliana* and shows an endopathogenic lifestyle. PLoS One.

[52] Martin CJ, Evans WJ (1986). Phytic acid-metal ion interactions. II. The effect of pH on Ca(II) binding. J. Inorg. Biochem..

[53] Kim NH, Rhee MS (2016). Phytic acid and sodium chloride show marked synergistic bactericidal effects against nonadapted and acid-adapted *Escherichia coli* O157:H7 strains. Appl. Environ. Microbiol..

