# Supplementary material for: Transcriptomic Approach for Understanding the Adaptation of Salmonella enterica to Contaminated Produce
Source: J Microbiol Biotechnol. 2020 Aug 21;30(11):1729–38. doi: 10.4014/jmb.2007.07036 (PMC9728351; doi:10.4014/jmb.2007.07036)
Supplement: Supplementary file 1 [file JMB-30-11-1729-supple1.pdf]

J. Microbiol. Biotechnol.
https://doi.org/10.4014/jmb.2007.07036

# Supplementary Data

## Table S1. Primers used in qRT-PCR

| Primer | Sequence (5′ →3′ ) |
| --- | --- |
| terB-RT-F | GTA AAA GGT GCC ATT AAC TCT G |
| terB-RT-R | GTC ACT CGA TAC GGC AAT AC |
| FORC38_1138-RT-F | CTGACAAAAGAGAAGCCTGCAA |
| FORC38_1138-RT-R | CATGCCTGCAAAGCCTGATT |
| terD-RT-F | TCGATGCTGGCTGCGTATTA |
| terD-RT-R | TATCGCCACTGTGCACAACA |
| fliL-RT-F | GCGATTAACAAAAAGAGCAAACG |
| fliL-RT-R | TGCTGCATACGCCAGTAGCTAT |
| fliT-RT-F | GTCGCCGGATACATCAAACA |
| fliT-RT-R | TTTTTGGCGGGTGGATTGT |
| fliC-RT-F | TGTCGCTGTTGACCCAGAATAA |
| fliC-RT-R | TCTTTCGCGCTGTTGATACG |
| motA-RT-F | TCCCTTGAACGCGATATTGAA |
| motA-RT-R | CAGGCGCAGATAATCGACAA |
| flgG-RT-F | TCGGTCGTCGATACTGCTTTAC |
| flgG-RT-R | AGGGTATGTCGAAACGTCGAA |
| flgF-RT-F | GCAGTTAACCATTCAGGGACATC |
| flgF-RT-R | GTCTGCCGCAATGGTGATTT |
| flgB-RT-F | CGCGCAACGTCAGGAAATAT |
| flgB-RT-R | CCACGCACCATCACCTTTTT |
| entA-RT-F | ATGCCGAACAGCAGCGTATT |
| entA-RT-R | TCGGAGGCAAGAAACAAAATG |
| entB-RT-F | ACTGGATATCCCGACCAACAAA |
| entB-RT-R | GCCCCAAAAGCTGACAAAGTAA |
| entE-RT-F | CTTTGACGCCAACGGTTTTT |
| entE-RT-R | GCCCCGATTGATCTGATCTTT |
| entC-RT-F | CGACTGCTGGCTTCACAAAA |
| entC-RT-R | ACTGCGGAGAGGAAGGTAATTG |
| argI-RT-F | TCTTTCGAAGTTGCCGCATA |
| argI-RT-R | ACGCGGGCAGTATCTTTGAT |
| FORC38_4149-RT-F | TCGTCCCAATCTCAGCCTAAA |
| FORC38_4149-RT-R | GTGTTGGCAAAAATGTCATGCT |
| FORC38_4150-RT-F | AAA CGC ATT GTG GAA AAC GAT G |
| FORC38_4150-RT-R | TGG TAG CCA TCG GCT TTT TC |
| FORC38_4151-RT-F | GCGGAAATCCAACACCTCAT |
| FORC38_4151-RT-R | TCAGCGCGATGTTTTTGC |

| | |
|---|---|
| ulaE-RT-F | CCGCTCGGCATCTATGAAAA |
| ulaE-RT-R | TCCACCGACATTTCAACGAA |
| ulaD-RT-F | TTAAACGGCTGTCCGATATGG |
| ulaD-RT-R | CCCGCGATAAAGACATGGAT |
| ulaC-RT-F | GCAGTTTGGCCCCTATTTTG |
| ulaC-RT-R | AACTCCAGCGGCTTTTTCAG |
| ulaB-RT-F | ACCGTGACCGGCAACAAATA |
| ulaB-RT-R | ACGTCTTGCGGGAAATGTTC |
| ulaG-RT-F | CAT GGA TTC TGA GCA CGT TTC |
| ulaG-RT-R | TTT AGC CAG ATA CCG GTA CAG |
| mgtC-RT-F | CGGCTTAGGGCAGTTCAAAA |
| mgtC-RT-R | CAGCGGATATTGGGAGTTGATT |
| sitD-RT-F | TCGGAAGTGCATCTGGATCA |
| sitD-RT-R | GATCTTTCCATTTCAGCCCAAT |
| sitC-RT-F | TTGGCAGCCTGACCAGTTTT |
| sitC-RT-R | GGCCGTGATGAACAGTAAGGTT |
| sitB-RT-F | GGCCGTGATGAACAGTAAGGTT |
| sitB-RT-R | TCGCGCTTCGGTTTTTACAT |
| sitA-RT-F | AACGCCCGGCGATATTAAAC |
| sitA-RT-R | GCCGGAAAGGTGCTGATAAA |
| mntH-RT-F | TGGGTACAGGCGGAAATCAT |
| mntH-RT-R | ACCGCGCCCTGTAATAAAGA |
| trpD-RT-F | TGAAAAATCCGGTGCTAATGC |
| trpD-RT-R | CCCAGACAAATGCCGATGAT |
| trpE-RT-F | AAGAGCGGGAGGCTATGTTTT |
| trpE-RT-R | AAAGCAGTAGTCCGGGCAGTTA |
| phoP-RT-F | CCGCAACCACCTACAAATACTG |
| phoP-RT-R | ATTCCCTTTCCTGGCGTCAT |
| thiC-RT-F | TCCCAACTCGAAACGCATCT |
| thiC-RT-R | TCTCTTCAAACTGCGGGTTGT |
| thiE-RT-F | GACAGCGTTGCATGGATTGA |
| thiE-RT-R | TAACATCCGCTTCCACCTCTTC |
| thiF-RT-F | CGCGATTTTATGCGCTACAG |
| thiF-RT-R | AACCCAATCCGCCTAAACCT |
| thiS-RT-F | TGCTCGCTACGCTTAATCAG |
| thiS-RT-R | CTGAAAAAGCAGGATCTGGTC |
| thiG-RT-F | CGTGCGATGCTGGAGATTATT |
| thiG-RT-R | TGTTAACCAGCACCGCATCA |
| thiH-RT-F | TCAAACCTCTGTGCCAACGA |
| thiH-RT-R | AGCATCGCACTCCCTTTGAA |

| | |
|---|---|
| nirD-RT-F | GGCAAAACATCTGCAAAATCG |
| nirD-RT-R | ATCGCTATGGTAAGGACGGAAA |
| nirB-RT-F | TAAATCCGACGCTAGCCTGTTC |
| nirB-RT-R | GGTGTGGTGAGAGAAGTAGGATGA |
| napG-RT-F | CCCTGCGAAATGTGTGAAGA |
| napG-RT-R | GAGTCATCAATGGAGGCGATATC |
| napH-RT-F | ACGCCGTATCAGCCAGTTTATG |
| napH-RT-R | GGAGGGTATCGAACAGCAAACTA |
| narJ-RT-F | CCTTGATCGGCGAAGAATTT |
| narJ-RT-R | CAGCGATAAAGTGGCTGAAAAA |
| narI-RT-F | ACCCGACCAGCTTCATCATT |
| narI-RT-R | ATTCTCTCGCTGCTGGTGATTC |
| cbiC-RT-F | GGCCATTATCAAGCGGGTTAT |
| cbiC-RT-R | TGGCGTAACGCATCACAAAG |
| cbiD-RT-F | ATG GTA TTG CGT CAA CAT CT |
| cbiD-RT-R | CAT CTT TAC GAA TTG CGG CTA |
| rpmB-RT-F | ACGCACTGAACGCGACTAAA |
| rpmB-RT-R | AGATACACGCAGGGTGACAAAA |
| rpmG-RT-F | TCTTCTGCTGGTACTGGTCACTTC |
| rpmG-RT-R | TGACGGACAACTGGATCGAA |
| rplC-RT-F | AATACACCGTAGGTCAGAGCATCA |
| rplC-RT-R | CTTAACGGTACCAGCGAAACCTT |
| rplD-RT-F | ACTACCTTCGGTCGTGATTTCAA |
| rplD-RT-R | TGGCGCCACGGTTTTTTA |
| rpsP-RT-F | AAAGCGTCCGTTCTACCAGGTT |
| rpsP-RT-R | GCGGGTGCCTTCTTCTTTTT |
| rimM-RT-F | TGACTATCAGCCCTGGTTTATCC |
| rimM-RT-R | ATCTCGATCGTCAACGCCTTT |
| trmD-RT-F | TCAGACCGAAATTGACGAAGAA |
| trmD-RT-R | TCGATTGCTGATGCCTCATG |
| tsf-RT-F | TGGCAAAATCACTGACGTAGAAG |
| tsf-RT-R | GACGCGACGAATGTTGATGT |
| spaP-RT-F | AAACACGTTGATGAAGGTCTGGAT |
| spaP-RT-R | TTCAGTTGCGCGTTTTCAAA |
| sipB-RT-F | CAGCGAAGGGCAATTGACAT |
| sipB-RT-R | ACTCAATCATCGCCTGCCATA |
| sipC-RT-F | AGGCTGATAGCAAACTGTCTGGTA |
| sipC-RT-R | TACCGGACAACGCATTCATC |
| sicP-RT-F | TGGTGAACTGGCTGCGAATA |
| sicP-RT-R | TTGTCAGATCGGTAATTGCATGT |
| iagB-RT-F | CTTGGCCTGATGCAAATTAACA |

| | |
|---|---|
| iagB-RT-R | CGCCCACAATGACAGAAATG |
| prgH-RT-F | TGGACCATGGCGGAGTAAAT |
| prgH-RT-R | TGCACCGAACGAGATTCAGA |
| prgI-RT-F | GGTAACAGAGGCGCTGGATAAA |
| prgI-RT-R | TGCGCGTTACGGTACAAGTTATA |
| ssaL-RT-F | TTTACTGGAACTTGGCGAAGTG |
| ssaL-RT-R | ACTGCGATAAGGGCATTTCATC |
| ssaK-RT-F | GCTGGAAAAAATGGAAGTGGAA |
| ssaK-RT-R | CTGCGTGATCGACCAATGAA |
| ssaJ-RT-F | TGCGCTACCGACTTATGATGA |
| ssaJ-RT-R | TTACCCGAAAGGCCTCCATAT |
| ssaI-RT-F | AACCGAGCCAGGAGCAAATT |
| ssaI-RT-R | TGGTCAACATAACCTGCGGTAA |
| sseE-RT-F | TTATCGCCTTACCTGGTGATCTATC |
| sseE-RT-R | ACTGCGGGTGATGCACAAATA |
| sseC-RT-F | TTAAAGCCGGAGCCGAAA |
| sseC-RT-R | CGACGGCTTCACAACCAAAT |
| sseA-RT-F | AAGGCTGCGTTTAGTGAATATCG |
| sseA-RT-R | CCCTTTCAGCAAGCTGTTGACT |
